# Echinacoside exerts anti-tumor activity via the miR-503-3p/TGF-β1/Smad aixs in liver cancer

**DOI:** 10.1186/s12935-021-01890-3

**Published:** 2021-06-10

**Authors:** Wen Li, Jing Zhou, Yajie Zhang, Jing Zhang, Xue Li, Qiao Yan, Jiabing Han, Fangdi Hu

**Affiliations:** School of Pharmacy, Lanzhou University, No. 199 Donggang West Road, Chengguan District, Lanzhou, Gansu 730000 China

**Keywords:** Echinacoside, Liver cancer, Apoptosis, MiR-503-3p, TGF-β1/smad

## Abstract

**Background:**

Echinacoside (ECH) is the main active ingredient of *Cistanches Herba*, which is known to have therapeutic effects on metastatic tumors. However, the effects of ECH on liver cancer are still unclear. This study was to investigate the effects of ECH on the aggression of liver cancer cells.

**Methods:**

Two types of liver cancer cells Huh7 and HepG2 were treated with different doses of ECH at different times and gradients. MTT and colony formation assays were used to determine the effects of ECH on the viability of Huh7 and HepG2 cells. Transwell assays and flow cytometry assays were used to detect the effects of ECH treatment on the invasion, migration, apoptosis and cell cycle of Huh7 and HepG2 cells. Western blot analysis was used to detect the effects of ECH on the expression levels of TGF-β1, smad3, smad7, apoptosis-related proteins (Caspase-3, Caspase-8), and Cyto C in liver cancer cells. The relationship between miR-503-3p and TGF-β1 was detected using bioinformatics analysis and Luciferase reporter assay.

**Results:**

The results showed that ECH inhibited the proliferation, invasion and migration of Huh7 and HepG2 cells in a dose- and time-dependent manner. Moreover, we found that ECH caused Huh7 and HepG2 cell apoptosis by blocking cells in S phase. Furthermore, the expression of miR-503-3p was found to be reduced in liver tumor tissues, but ECH treatment increased the expression of miR-503-3p in Huh7 and HepG2 cells. In addition, we found that TGF-β1 was identified as a potential target of miR-503-3p. ECH promoted the activation of the TGF-β1/Smad signaling pathway and increased the expression levels of Bax/Bcl-2. Moreover, ECH could trigger the release of mitochondrial Cyto C, and cause the reaction Caspases grade.

**Conclusions:**

This study demonstrates that ECH exerts anti-tumor activity via the miR-503-3p/TGF-β1/Smad aixs in liver cancer, and provides a safe and effective anti-tumor agent for liver cancer.

## Introduction

Liver cancer is one of the most common malignant tumors with a high incidence rate, and the incidence of liver cancer has been growing rapidly [1]. Hepatectomy is the best way to treat liver cancer for patients at early stages [2]. However, most patients have recurrence and metastasis after surgery. The 5-year survival rate is only about 3–5% [2]. Therefore, novel therapeutic approaches and agents are urgently needed.

In the past few years, cancer drug development and research phytochemicals have started to pay attention to ethnopharmacology [3, 4]. A large number of clinical and experimental studies have shown that medicine monomers can inhibit the proliferation, metastasis of hepatocellular carcinoma cells, induce apoptosis and differentiation of cancer cells [5]. *Cistanches Herba* is a plant belonging to the family of plants, which is a traditional medicine in China and is known as “desert ginseng” [6]. *Cistanches Herba* is rich in types of chemical components, and researchers at home and abroad have isolated more than a hundred compounds from it, including phenylethanol glycosides, lignans, cycloaliphatic terpenes, sugars, amino acids, etc. [7]. Echinacoside(ECH) is the main component of *Cistanches Herba* and is also generally considered as its main active ingredient [6]. In recent years, studies have shown ECH may be a new treatment option for aggressive or widely metastatic tumors [8]. Studies have found that ECH can inhibit pancreatic cancer, leukemia, gastric cancer, endometrial cancer and many other tumors [9]. However, there is no report about the effect of ECH on the progression of liver cancer.

MiRNAs play pivotal roles in the post-transcriptional regulation of gene expression (10). Extensive studies have shown that miRNAs are closely related to liver cancer [11] and Multiple Sclerosis. MiR-503-3p is a newly discovered miRNA that is involved in proliferation, migration, and invasion of tumor cells [12]. However, its function in liver cancer is unclear. At present, the research of miRNA downstream target genes is in a relatively mature stage, but many target gene loci remain undiscovered. The TGF-β1/Smad3 signal transduction pathway is particularly important for tumorigenesis and development [13]. It was found that the TGF-β1/Smad3 pathway was activated in hepatoma model, suggesting that the TGF-β1/Smad3 pathway played a significant role in the development of liver cancer [14]. The high expression of TGF-β1/Smad3 pathway is considered to be one of the key reasons for the occurrence of liver cancer [15]. In order to further investigate the underlying mechanism mediated the effects of ECH on the liver cancer, miRNA/ TGF-β1/Smad3 pathway was explored.

In this study, we evulated the effects of ECH on the progression of liver cancer cells, and found that ECH exerted an anti-tumor activity via the miR-503-3p/TGF-β1/Smad aixs in liver cancer, and provides a safe and effective anti-tumor agent for liver cancer.

## Methods and materials

### Materials

**Fig. 1:**
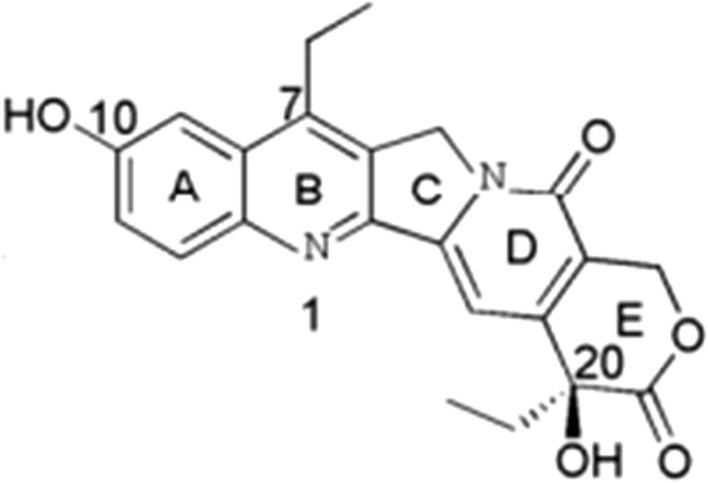
The structural formula of Echinacoside

Echinacoside (ECH) (purity > 98%, monohydrate) was obtained from Hetian Dichen sasheng Pharmaceutical Development Co., Ltd (Xinjiang, China). The structural formula was shown in Fig. 1.

### Cell culture and transfection

Human liver cancer cell lines Huh7 and HepG2 were purchased from FENGHUISHENGWU (Hunan, China). Cells were cultured in DMEM medium supplemented with 10% fetal bovine serum (FBS, Gibco, USA) at 37 ℃ in a 5% CO_2_ cell incubator. MiR-503-3p mimic and miR‑NC were obtained from Shanghai Gene Pharmaceutical Co., Ltd. (Shanghai, China). All transfections were performed using Lipofectamine 2000 reagent (Invitrogen) following manufacturer’s instructions. After an additional 24–48 h, the transfected cells were collected and processed for further studies.

### Luciferase reporter assay

Total RNAs were extracted from cells, and TGF-β was amplified using the WT and MUT primers. A fragment of TGF-β1 was inserted into the pGL3-Bashc luciferase reporter vector named TGF-β-WT-Luc. The binding region of β1 and miR-503-3p was mutagenized to form a mutant plasmid designated as TGF-β1-Mut-Luc. TGF-β1-WT-Luc and TGF-β1-Mut-Luc were co-transfected into cells with miR-503-3p mimic and pRLTK. Cells were collected 6 h after transfection. Dual-Luciferase Reporter Assay Kit (Promega, Madison, WI, USA) was used to determine luciferase activity.

### Cell growth assay

Huh7 and HepG2 cells (2 × 10^5^) were placed in 96-well plates. Cells were then treated with ECH for 24, 36 and 48 h. After that, 10 mL MTT solution was added to each well and the cells were incubated for 4 h. After that, 150 μL of DMSO was added to the wells. And the absorbance at 570 nm was measured by an ELISA reader (Aoweiya, Beijing, China).

### Colony formation

Single cell suspension was prepared from the transfected cells and cultured in an incubator for 7 days. After discarding the medium, cells were stained with Wright’s stain for 5 min, and then stained with Giemsa dye solution and Sorensen phosphomolybdate buffer solution (1:9) for 10 min. After washing, colonies were counted under a microscope.

### Transwell assay

Migration and invasion assays were performed in Transwell chambers (Costar, Badhoevedorp, The Netherlands) coated with or without Maribel (BD Biosciences) on the upper surface of the 8-μm (pore size) membrane. For migration assay, transfected Huh7 and HepG2 cells were re-suspended in 200 µL of serum-free DMEM medium and seeded in the upper chambers. For invasion assay, Matrigel (30 μg/well) was pre-coated on the membranes of the upper chambers and other steps were the same as migration assay. After incubation of 24 h, cells had migrated or invaded through the membrane to the lower surface were fixed with ethanol and stained with 0.2% crystal violet. Finally, cells were observed and counted in five random fields using a microscope.

### Cell cycle analysis

Huh7 and HepG2 cells were seeded in 6-well plates at a density of 1 × 10^5^ cells/mL. Cells were collected after treated with ECH for 0, 24, 48 h. These cells were then added with 1 mL of ice precooled 75% ethanol, fixed at 20 ℃ for 2 h. After that, cells were added with PI staining solution and incubated in dark for 15 min. Cell cycle was then analyzed by flow cytometry.

### Apoptotic cell analysis

Cells were plated into 6-well plates at a density of 5 × 10^5^ cells/well. After collection, cells were centrifuged and 195 μL of Annexin V-FITC binding solution was added to resuspend the cells. Next, 5 μL Annexin V-FITC and 10 μL propidium iodide staining solution were added. Cells were incubated in the dark and then placed in an ice bath.

### Quantitative reverse transcription PCR (qRT-PCR)

Total RNAs were extracted by TRIzol reagent (Haigene, Haerbin, China). The viiA™ 7 real-time PCR system was used for qRT-PCR. RNA was reverse transcribed into cDNA using the BeyoRT™ First Strand cDNA Synthesis Kit (Beyotime Institute of Biotechnology). U6 was used as an endogenous control [16]. The primer sequences were as follows:

miR-503-3p: forward: 5′-CTGATGGTTAAGAGAATGT-3′,

miR-503-3p: reverse: 5′-GTCCTTGGACATCCGGGCCG-3′,

U6: forward: 5′-GACAGATTCGGTCTGTGGCAC-3′,

U6: reverse: 5′-GATTACCCGTCGGCCATCGATC-3′.

### Western blot analysis

The transfected cells were collected and total proteins were extracted. Protein concentrations were determined using the BCA Protein Assay Kit. Protein samples were separated by 10% SDS-PAGE and transferred onto a polyvinylidene fluoride membrane. The membrane was then incubated with anti-TGF-β1 (1:1000, Shifeng, Shanghai, China), SMAD3 (1:1000, Shifeng, Shanghai, China), SMAD 7 (1:1000, Shifeng, Shanghai, China), Bcl-2 (1:1000, Shifeng, Shanghai, China), Bax (1:1000, Shifeng, Shanghai, China), Cyto C (1:1000, Shifeng, Shanghai, China), caspase-3 (1:1000, Shifeng, Shanghai, China), caspase-8 (1:1000, Shifeng, Shanghai, China) and anti-β-actin antibody (1:1000, Shifeng, Shanghai, China) overnight. Then the membrane was incubated with anti-rabbit secondary antibody (1:1000, Shifeng, Shanghai, China) [17].

### Statistical methods

The data were presented as the mean ± standard deviation (SD). All statistical analyses were performed with SPSS 13.0 software. Student’s t-test was used for comparisons. *p* value < 0.05 was considered significant.

## Results

### Effect of ECH on human liver cancer cell activity and proliferation

**Fig. 2:**
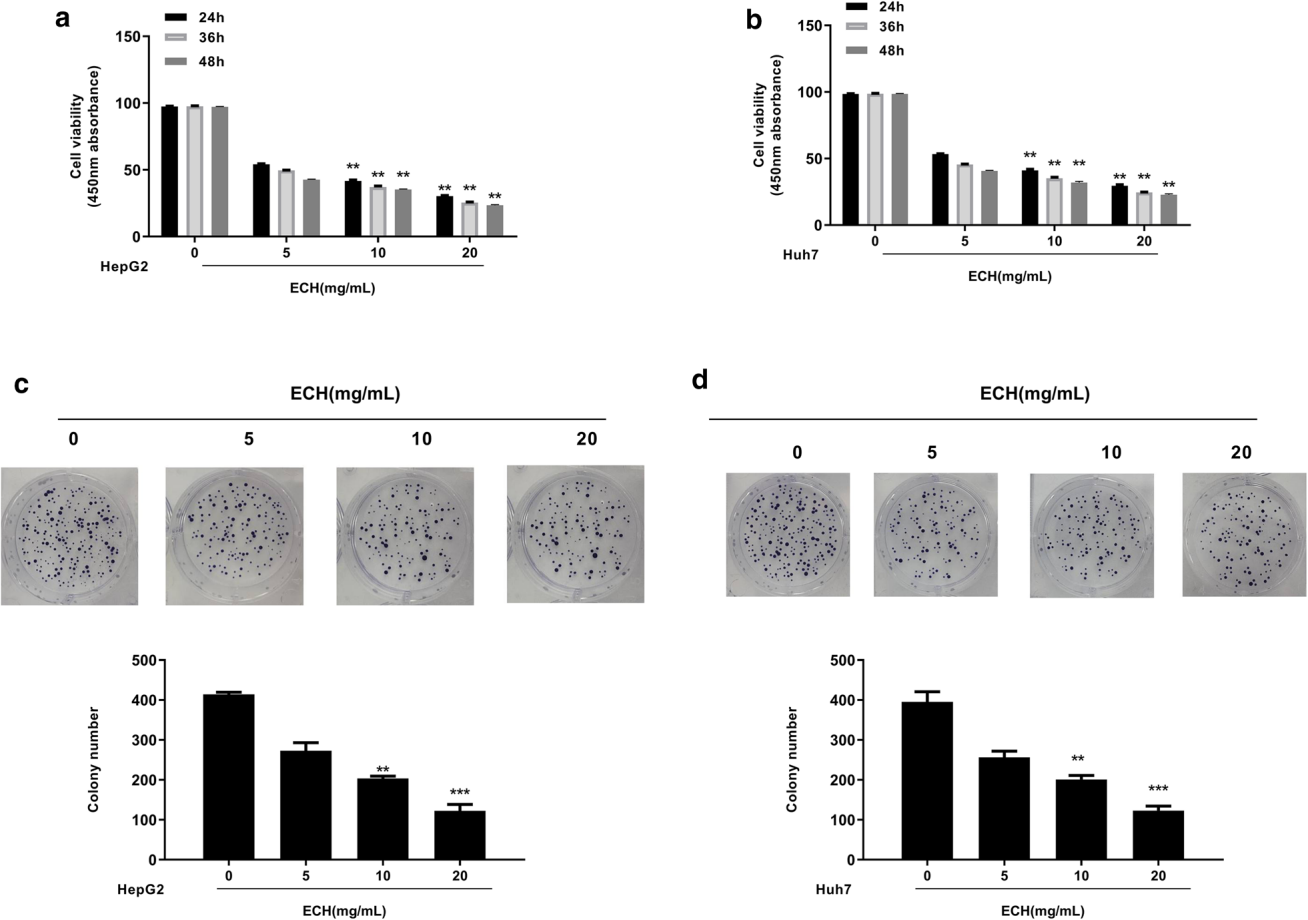
**a** Effect of ECH on cell viability of HepG2 cells at different dose and time of action. **b** Effect of ECH on cell viability of Huh7 cells at different dose and time of action. **c** Effect of ECH concentration on cell colony formation of HepG2 cells at different dose and time of action. **d** Effect of ECH concentrations on cell colony formation of Huh7 cells at different dose and time of action. N = 6, means ± SD, VS the 0 group, **p < 0. 01, *** *p* < 0.001

To investigate the effect of ECH on human liver cancer cell function, MTT assay was performed and the results showed that the cell viability of HepG2 (Fig. 2a) and Huh7 (Fig. 2b) was gradually decreased with the increase of dose and time of ECH treatment compared with the control group (*p* < 0.01). After 48 h exposure of ECH, the cell viability was the lowest, so 48 h exposure of ECH was selected for further experiment. Colony formation assay results showed that the number of colonies of HepG2 (Fig. 2c) and Huh7 (Fig. 2d) cells gradually decreased by treatment of ECH in a dose-dependent manner compared with the control group (*p* < 0.01, *p* < 0.001). These results indicate that ECH plays an important role in HCC cell activity and proliferation.

### Effects of ECH on human liver cancer cell migration and invasion

**Fig. 3:**
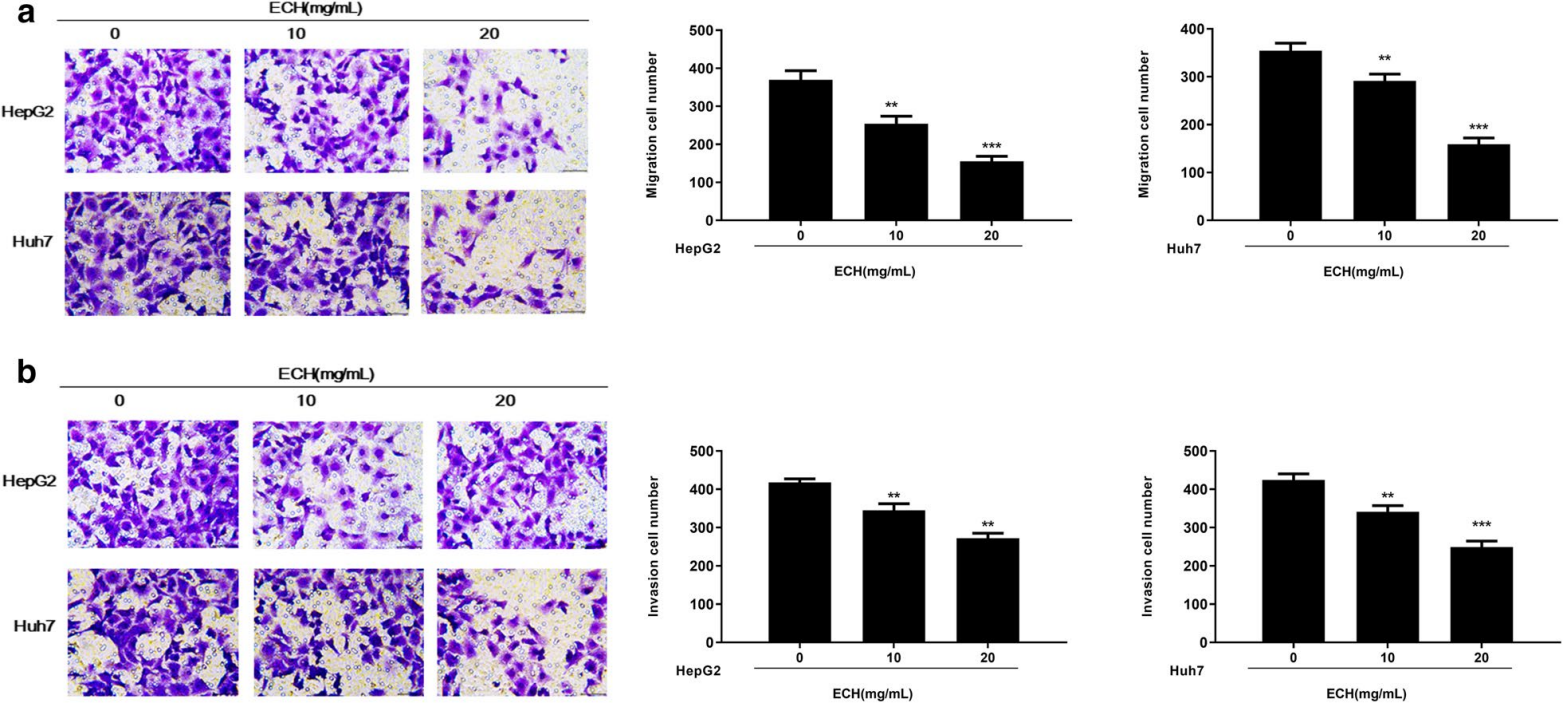
Effect of ECH on cell migration and invasion of HepG2 and Huh7 cells by Transwell assays. **a** Effect of ECH on migration of HepG2 and Huh7 cells. **b** Effect of ECH on invasion of HepG2 and Huh7 cells. N = 6, means ± SD, VS the 0 group, **p < 0. 01, ***p < 0.001

To further investigate the effect of ECH on human liver cancer cell function, cell migration and invasion were detected. As shown in Fig. 3a, b, compared with the group without treatment of ECH, the migration and invasion of Huh7 and HepG2 cells were significantly reduced after treatment of ECH in a dose-dependent manner (*p* < 0.01, *p* < 0.001), suggesting that ECH inhibited the migration and invasion of Huh7 and HepG2 cells.

### ECH regulated human liver cancer cell distribution and apoptosis

**Fig. 4:**
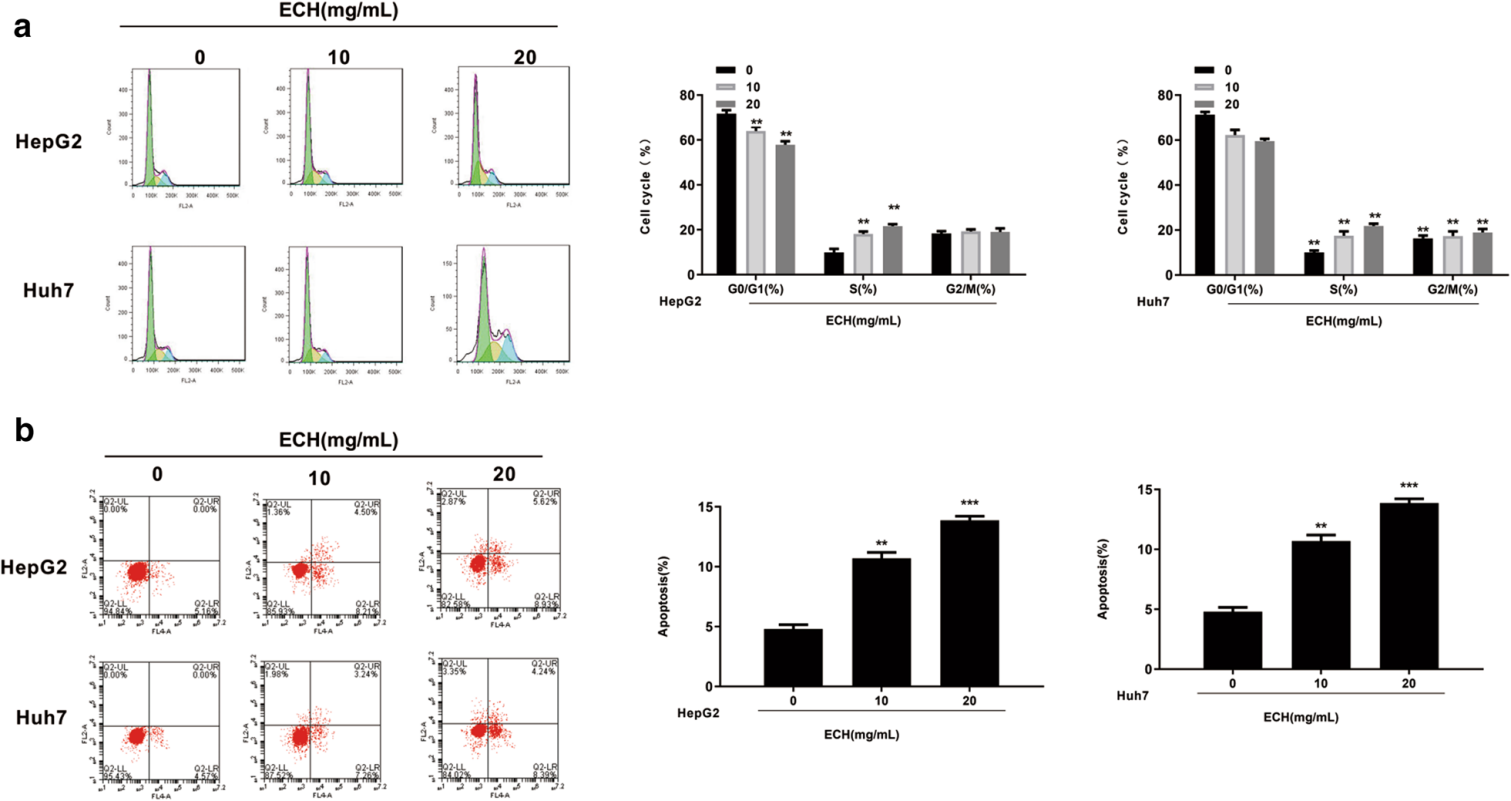
Effect of ECH on cell cycle and apoptosis of HepG2 and Huh7 cells by flow cytometry. **a** Effect of ECH on cell cycle of HepG2 and Huh7 cells. **b** Effect of ECH on apoptosis of HepG2 and Huh7 cells. N = 6, means ± SD, VS the 0 group, **p < 0. 01, ***p < 0.001

In order to explore the effect of ECH on the liver cancer cell apoptosis, flow cytometry was carried out. As shown in Fig. 4a, compared with the control group, the percentage of Huh7 and HepG2 cells in G0/G1 phase was significantly reduced after ECH treatment in a dose-dependent manner (*p* < 0.01), and the percentage of Huh7 and HepG2 cells in the S phase was gradually increased after ECH treatment in a dose-dependent manner (*p* < 0.01), but there was no change of the percentage of Huh7 and HepG2 cells in G2/M phase after ECH treatment (*p* > 0.05), indicating that ECH caused S phase arrest of Huh7 and HepG2 cells. In addition, with the increase of the dose of ECH, the apoptosis rate of Huh7 and HepG2 cells gradually increased compared with the control group (Fig. 4b, p < 0.01, *p* < 0.001). These results indicated that ECH induced the apoptosis of Huh7 and HepG2 cells.

### The effects of ECH on the TGF-β1/Smad pathway

**Fig. 5:**
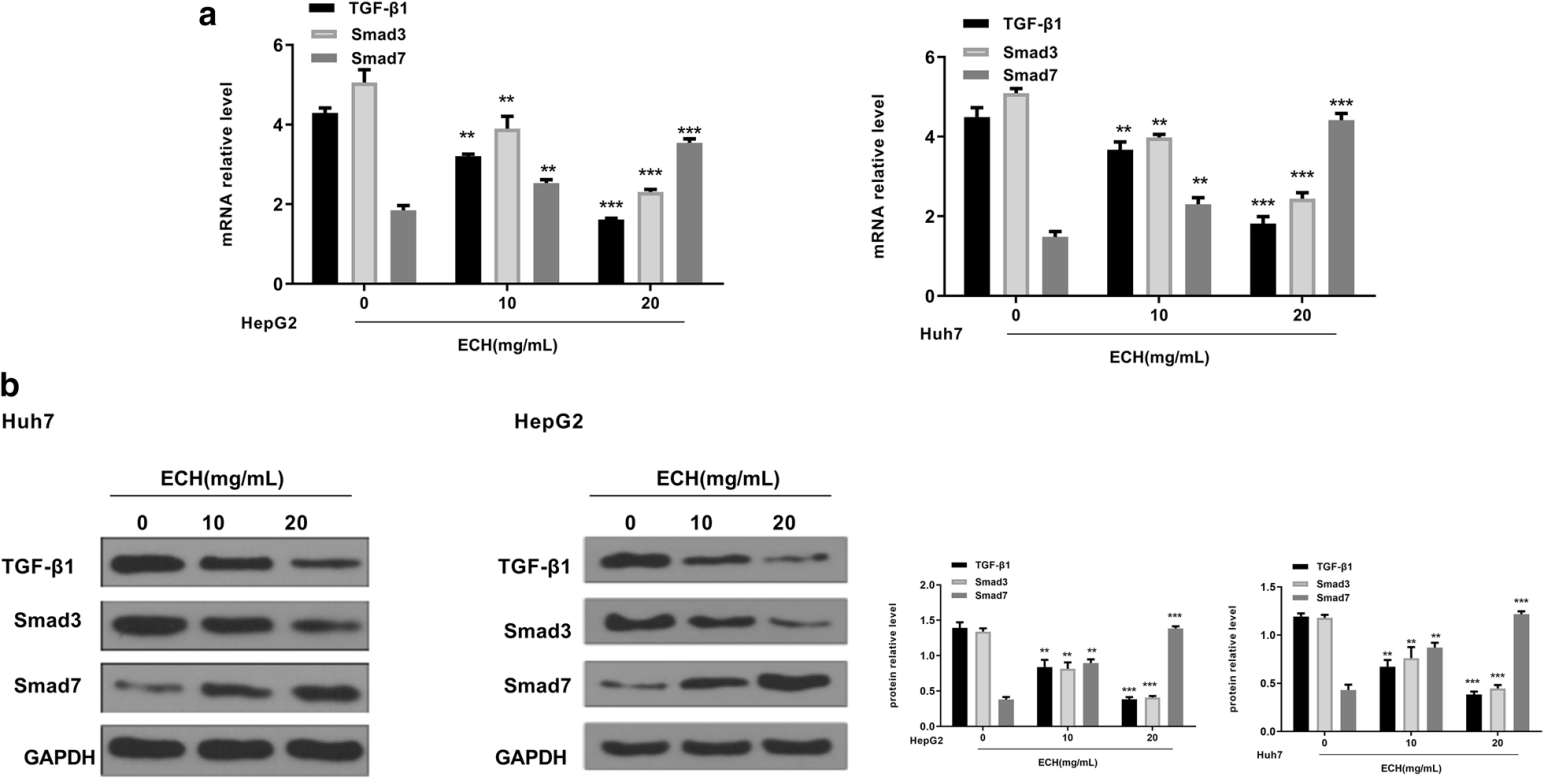
Effect of ECH on TGF-1/Smad pathways. **a** The mRNA levels of TGF-1, smad3 and smad7 in HepG2 and Huh7 cells. **b** The protein levels of TGF-1, smad3 and smad7 in HepG2 and Huh7 cells. N = 6, means ± SD, VS the 0 group, **p < 0. 01, ***p < 0.001

It’s known that TGF-β1/Smad signaling pathway is well involved in the cell apoptosis. In order to investigate the mechanism of ECH-induced apoptosis in HepG2 cells. The expression of TGF-β1/Smad signaling pathway was detected. Compared with the control group, we found that ECH treatment significantly reduced the expression levels of TGF-β1 and Smad3 at both mRNA (Fig. 5a) and protein (Fig. 5b) levels, and significantly up-regulated the expression of Smad7 in a dose-dependent manner at both mRNA and protein levels (*p* < 0.01, *p* < 0.001). These results indicated that ECH inhibited the activation of the TGF-β1/Smad signaling pathway.

### TGF-β1 was a direct target of miR-503-3p

**Fig.6:**
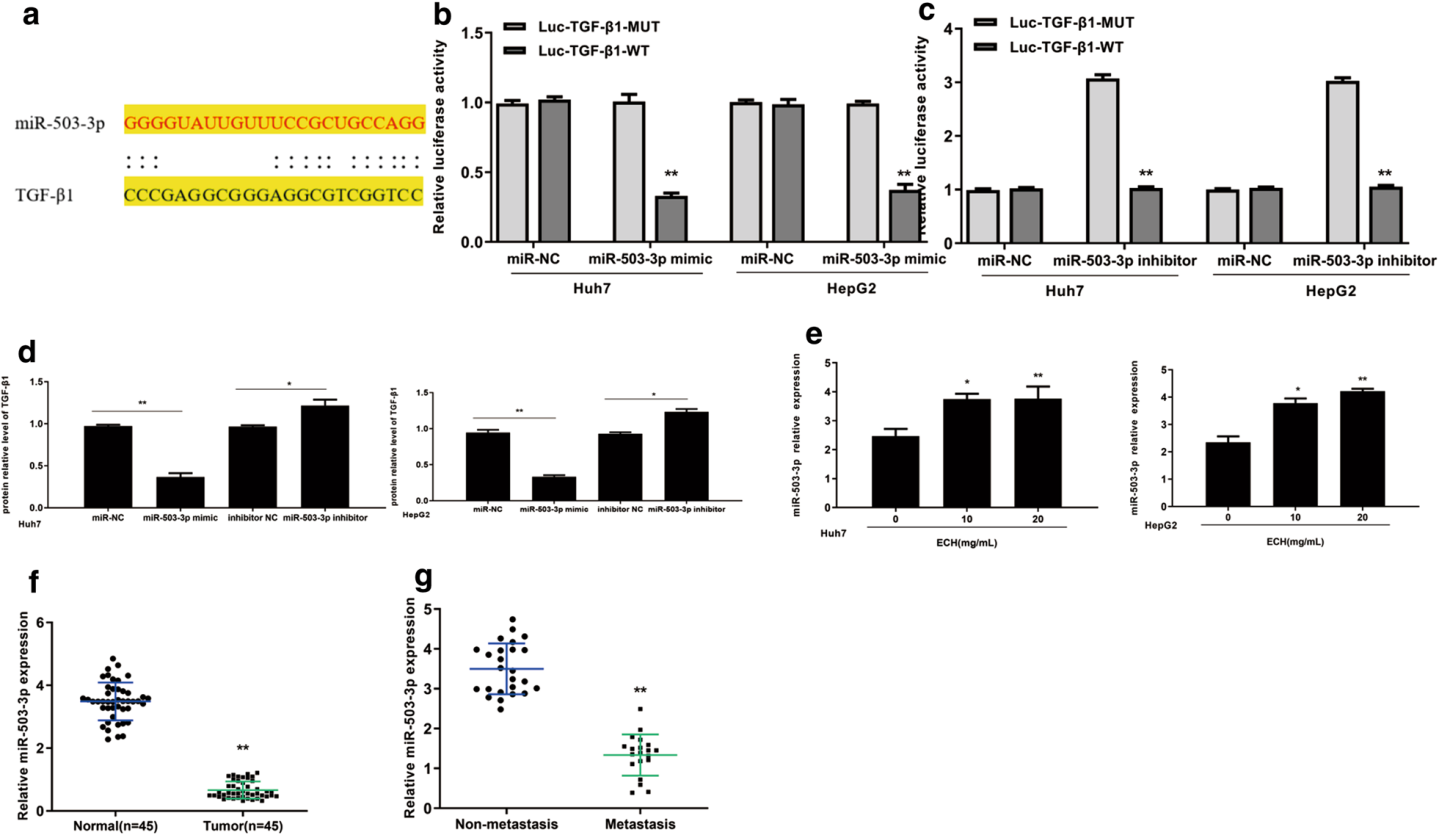
TGF-β1 was directly targets of miR-503-3p. **a** Putative targeting sites for TGF-β1 and miR-503-3p. **b** HepG2 cells and Huh7 cells were cotransfected with the miR-503-3p mimic and reporter plasmid containing the wild-type (WT) or Mut (MT) 3′-UTR of TGF-β1. A luciferase activity assay was subsequently performed. **c** HepG2 cells and Huh7 cells were cotransfected with the miR-503-3p inhibitor and reporter plasmid containing the wild-type (WT) or Mut (MT) 3′-UTR of TGF-β1. A luciferase activity assay was subsequently performed. **d** Protein expression levels of TGF-β1 after transfection with miR-503-3p mimic and inhibitor in HepG2 cells and Huh7 cells. **e** Expression levels of miR-503-3p was increased after exposure of ECH in HepG2 and Huh7 cells. N = 6, means ± SD, VS the 0 group, * *p* < 0. 05, **p < 0.01

In addition, the online prediction tool Starbase v2.0 (http://starbase.sysu.edu.cn) showed that TGF-β1 was a potential target of miR-503-3p (Fig. 6a). Luciferase reporter gene assay results showed that the luciferase activity of HepG2 cells and Huh7 cells co-transfected with miR-503-3p mimic and TGF-β1-WT was significantly reduced, but not the cells co-transfected with miR-503-3p mimic and TGF-β1-MUT (Fig. 6b, p < 0.01). Similarly, the luciferase activity of HepG2 and Huh7 cells co-transfected with miR-503-3p inhibitor and TGF-β1-MUT was significantly increased, but not the cells co-transfected with miR-503-3p inhibitor and TGF-β1-WT (Fig. 6c, p < 0.01). Furthermore, the expression levels of TGF-β1 were significantly reduced in miR-503-3p mimic group compared with that in the NC group (Fig. 6d, p < 0.01), but significantly increased in the miR-503-3p inhibitor group (Fig. 6d, p < 0.05). These results suggested that TGF-β1 was a direct target of miR-503-3p. To further investigate whether miR-503-3p mediated the effect of ECH on liver cancer, the expression of miR-503-3p was detected in liver cancer tissues and it showed that the expression levels of miR-503-3p were decreased in liver cancer tissues compared with that in normal liver tissues (Fig. 6f, p < 0.01). Moreover, the expression levels of miR-503-3p were further decreased in metastasis liver cancer tissues than that in nonmetastatic liver cancer tissues (Fig. 6g, p < 0.01). The expression levels of miR-503-3p in HepG2 and Huh7 cells were gradually increased after ECH exposure (Fig. 6e, p < 0.05, *p* < 0.01). Taken together, these results suggest that miR-503-3p/TGF-β1 may mediate the effect of ECH on the liver cancer.

### Effect of ECH on cytochrome C and caspases activities

**Fig. 7:**
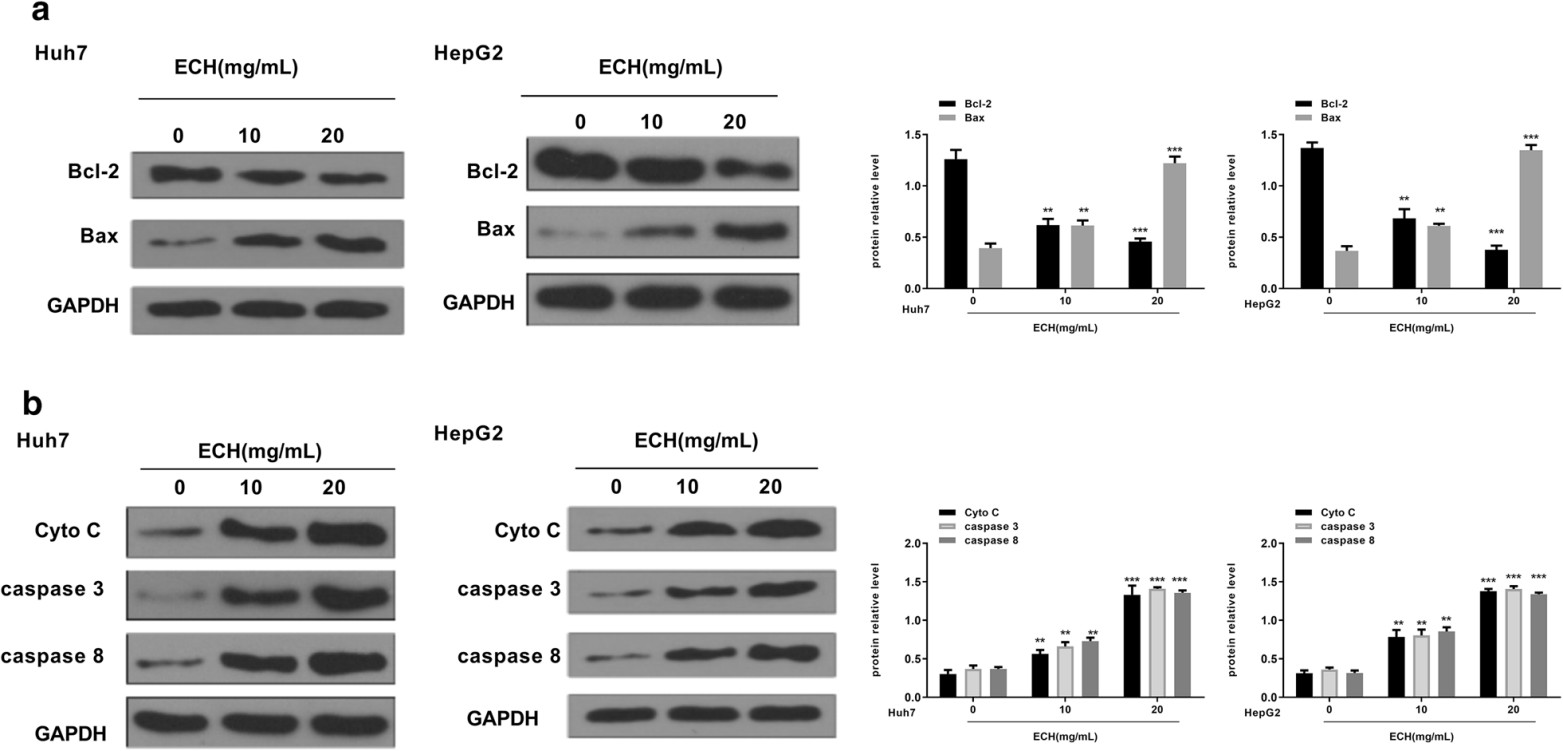
Effect of ECH on Bcl-2, Bax, cytochrome C, caspases-8 and caspase-3 in HepG2 cells. **a** Effect of ECH on Bcl-2 and Bax in HepG2 cells. **b** Effect of ECH on cytochrome C, caspases-8 and caspase-3 in HepG2 cells. n = 6, means ± SD, VS the 0 group, **p < 0. 01, ***p < 0.001

As shown in Fig. 7a, compared with the control group, we found that ECH treatment induced a significant decrease in the expression levels of Bcl-2 in a dose-dependent manner (*p* < 0.01, *p* < 0.001), while the expression levels of Bax were significantly increased in a dose-dependent manner (*p* < 0.01, *p* < 0.001). It was demonstrated that Bcl-2 and Bax were involved in ECH-induced apoptosis in HepG2 and Huh7 cells. In addition, the expression levels of Cyto C, caspase-8 and caspase-3 were significantly increased in HepG2 and Huh7 cells (Fig. 7b, p < 0.01, *p* < 0.001), indicating that the release of Cyto C and the activation of caspase-8 and caspase-3 were involved in ECH-induced apoptosis in HepG2 and Huh7 cells.

## Discussion

Surgical resection is the main treatment method for patients with liver cancer, and the recurrence and metastasis rate after liver resection is high [18, 19]. In this study, we found that ECH has a certain inhibitory effect on liver tumors, and provides a safe and effective anti-tumor agent for liver cancer. Clinical practice has demonstrated that traditional Chinese medicine can effectively alleviate the clinical symptoms of liver cancer patients [20]. For ECH, studies have shown that ECH has antioxidant, antidepressant, anti-inflammatory, antiviral, antitumor and antibacterial effect [21, 22]. In colorectal cancer, ECH can induce tumor cell apoptosis by up-regulating caspase-3 and cleavage DNA repair enzymes to induce DNA oxidation [23]. In this study, we found for the first time that ECH exerts anti-tumor activity via the miR-503-3p/TGF-β1/Smad aixs in liver cancer.

The intercellular phase of cell cycle of eukaryotic cells can be divided into G1, S, and G2. The regulation points of the cell cycle at different periods are regulated by different regulatory factors [24]. This study found that the percentage of Huh7 and HepG2 cells in G0/G1 and G2/M phase was reduced, and the percentage of cells in S phase was increased after ECH, indicating that ECH caused S phase arrest of Huh7 and HepG2 cells. Imbalance of apoptosis is one of the main mechanisms of human tumors. It has been reported that anti-tumor chemotherapeutic drugs generally induce apoptosis of cancer cells and control cancer progression. In our study, we also found that ECH could induce liver cancer cell apoptosis in a dose-dependent manner, suggesting that inducing cancer cell apoptosis may be one of the underlying mechanism of ECH on liver cancer.

MiRNAs play key roles in the regulation of HCC cell proliferation, cycle, apoptosis, migration through interactions with different signaling pathways and molecules [25, 26]. For example, it was found that miR-26a is highly expressed in normal liver cells, but it is significantly downregulated in liver cancer cells and inhibiting proliferation [27]. Overexpression of miR-503-3p induces apoptosis of lung cancer cells and inhibits cell invasion and migration [28]. In addition, a large number of studies have shown that the TGF-β1/Smad signaling pathway plays an pivotal role in the development of liver cancer [7]. Moreover, when liver cancer continues to progress, the expression of TGF-β1 is reported to be gradually increased [29]. In this study, we found that ECH down-regulated TGF-β1 and Smad3, and up-regulated Smad7, demonstrating that ECH inhibited the activation of the TGF-β1/Smad signaling pathway. In this study, we found that the expression of miR-503-3p was found to be reduced in liver tumor tissues, but ECH treatment increased the expression of miR-503-3p in Huh7 and HepG2 cells. In addition, we found that TGF-β1 was identified as a potential target of miR-503-3p. ECH promoted the activation of the TGF-β1/Smad signaling pathway and increased the expression levels of Bax/Bcl-2. These results suggest that miR-503-3p/ TGF-β1/Smad signaling pathway may mediate the effects of ECH on the liver cancer.It has been known that the increased Bax/Bcl-2 ratio may cause Cyto C in the mitochondria to be released into the cytoplasm. Cyto C interacts with Caspase-8 and forms an apoptotic body that then recruits and activates Caspase-3, which causes the Caspases cascade and promotes cell death [30]. In this study, we found that ECH reduced the expression of Bcl-2 and increased the expression of Bax, Cyto C, caspase-8 and caspase-3. These results suggest that ECH can repair mitochondrial injury to a certain extent.

## Conclusion

In this study, we found that ECH treatment inhibited the proliferation, invasion and migration, and induced the apoptosis of liver cancer cells. The underlying mechanism of ECH treatment on liver cancer cells was found to be related to the miR-503-3p/TGF-β1/Smad signaling pathway. This study will provide a certain experimental basis for the research of ECH on anti-tumor. It is worth noting that the present study is limited by the lack of animal experiments. Therefore, our future study will perform animal experiments to further confirm our findings.

## Data Availability

The data used and/or analyzed during the current study are available from the corresponding author on reasonable request.
